# A Salt Tolerance Evaluation Method for Sunflower (*Helianthus annuus* L.) at the Seed Germination Stage

**DOI:** 10.1038/s41598-020-67210-3

**Published:** 2020-06-30

**Authors:** Wenhui Li, Huizhen Zhang, Youling Zeng, Lijun Xiang, Zhonghua Lei, Qixiu Huang, Tianye Li, Fei Shen, Quan Cheng

**Affiliations:** 1 Xinjiang Key Laboratory of Biological Resources and Genetic Engineering, College of Life Science and Technology, Xinjiang University, Urumqi, 830046 China; 2 Institute of Economic Crops, Xinjiang Academy of Agricultural Sciences, Urumqi, China

**Keywords:** Plant breeding, Plant stress responses

## Abstract

Salinity is a major abiotic stress that affects plant growth and development and leads to crop yield loss. Many crop species are more sensitive to salinity stress at the seed germination stage than at other developmental stages. Some studies have shown that sunflower is tolerant to salinity to a certain degree. However, no systematic screening data for sunflower germplasms are available for salinity stress. In this study, 552 sunflower germplasms with different genetic backgrounds were evaluated for salt tolerance. Among them, 30 and 53 sunflower germplasms were identified as highly salt-tolerant and salt-tolerant germplasms, respectively, while 80 and 23 were grouped as salt-sensitive and highly salt-sensitive materials, respectively. Of all the traits tested, the germination index and the germination vigor index were the two most reliable traits, showing the highest correlation with salt tolerance during the seed germination stage of sunflower. Thus, a highly efficient and reliable method for evaluating salinity tolerance of sunflower seed germination was established. These results provided a good foundation for studying salt-tolerance mechanisms and breeding highly salt-tolerant sunflower cultivars.

## Introduction

Salinity is a major abiotic stress that affects plant growth and development, thus resulting in crop yield loss. High salt stress disrupts homeostasis of water potentials and ion distributions, leading to molecular damage, reduced growth and even cell death^1^. Worldwide, more than 800 million hectares of land are affected by salt, equivalent to 6% of the total land area^2^, affecting more than 20% of today’s agriculture^3–7^. A more efficient way to use land with saline soil is to screen existing germplasms and develop new crop varieties with high tolerance to salinity stress^8^. Sunflower (*Helianthus annuus* L.) is the fifth most widely grown edible oil crop in the world, and its planting area exceeds 22.9 million hectares across 60 countries with a total value of over $40 billion annually^9^. The development and breeding of salt-tolerant sunflower varieties are very necessary and have enormous economic potential^10–13^. Thus far, crossbreeding is still a commonly used breeding method; however, the traditional breeding methods focus on screening germplasms with desired traits, such as those with high tolerance to salinity.

Sunflower is a crop with moderate salt- tolerance^14^. However, salinity stress is still a major constraint in sunflower breeding owing to inadequate rainfall failing to leach salt from the root zone and high evapotranspiration often exceeding rainfall^15^. Generally, the ability of a crop to survive and grow under saline conditions depends on its salt tolerance, which can vary among different crops and growth stages^16^. Seed germination is the first stage of crop growth and development during the plant life cycle. Thus, high germination ability of crops in saline soil is necessary for later growth and development. It has been reported that salt stress can lead to a significant reduction in germination rate, as it reduces the ability of plants to uptake water from the soil, resulting in the growth inhibition and yield loss. Accordingly, to accelerate salt tolerance breeding of sunflower, an effective method to evaluate and obtain salt-tolerant germplasms at the germination stage or other growth stages in sunflower is urgently needed. In the current study, we establish a high-quality reference for the screening and evaluation of salt-tolerant sunflower germplasms at the seed germination stage.

Evaluation methods for the screening of salt-tolerant germplasms have been developed in various plant species at the seed germination stage. However, the methods are inconsistent among different crops^2,17^. In oilseed rape, the fresh weight of shoot is an effective screening feature for salt tolerance at the germination stage^17^, and among the traits examined in a recent study, the germination index of sweet sorghum (*Sorghum bicolor* (L.) Moench.) had the highest correlation with salt tolerance^2^. Salt tolerance is a complex quantitative trait, and the measurement of a single trait poorly reflects the tolerance of plants to stress. Membership function analysis is used to integrate more traits in order to screen and evaluate plant tolerance germplasms in various plant species^18,19^. The drought tolerance of wheat resources can be divided into five distinct grades according to mean and standard deviation^19,20^. Wu *et al*. applied multiple regression to establish a quantitative evaluation model for salt tolerance of rapeseed inbred lines at the germination stage^17^. For sunflower, there is currently no reliable method for evaluating and/or screening germplasms with salinity tolerance. A recent study showed that seed shape is a potential predictor of salt tolerance in sunflower^21^. Two additional studies have investigated the salt tolerance index of mature sunflower plants, but we considered it was also unreliable to provide an effective evaluation with insufficient samples^22,23^. In addition, there seems to be little research on salt tolerance at the seed germination stage in sunflower.

In this study, the optimal concentration for salt tolerance screening of sunflower germplasms was first determined. Subsequently, 552 sunflower germplasms (inbred lines) were phenotyped for a variety of traits at the seed germination stage, and a quantitative evaluation model developed from multiple regression analysis was established for salt tolerance. Besides, the germination index and the germination vigor index were found to be the two most reliable traits for salt tolerance of sunflower at the germination stage based on correlation analysis. These results improve the current basis for sunflower breeding and the exploration of salt tolerance mechanisms.

## Materials and methods

### Plant materials

The seeds of 729 sunflower germplasms (maintained by the Institute of Economic Crops, Xinjiang Academy of Agricultural Sciences, China) were collected from different regions of China and other countries. These germplasms had different genetic backgrounds and were labeled with numbers that described each line. The first two numbers corresponded to harvest year of resource seeds, while the third represented the various types of lines; in the third numbers, 1 or 2 indicated restorer lines, and 6 indicated sterile lines or maintainer lines. A pre-experiment was first conducted to ensure the viability of all seeds based on high germination rates (>99%). Only such high-quality seeds were used in subsequent experiments to evaluate salt tolerance at the germination stage.

### Determination of salt stress concentration

The ZSADT variety of oil sunflower is widely cultivated as a major parental line for sunflower breeding in Xinjiang, China, and it has some excellent agronomic traits, such as early maturity, dwarf phenotype, lodging resistance and large floral disc size. It was used to determine the optimal salt concentration for the salt-tolerant screening of sunflower germplasms and a method of salt tolerance evaluation was developed at the seed germination stage. NaCl concentrations in the test included 25, 50, 75, 100, 150, 200, 250, 300, and 400 mM NaCl. All seeds were shelled, sown in 9-cm-diameter Petri dishes and cultured in an incubator at 28 °C/18 °C (day/night) with 16 h light/8 h dark. Seeds treated with distilled water (0 mM NaCl) served as controls. Four biological replicates were designed per treatment in one experiment. When the radicle length reached half of the seed length, the seed was considered to be germinated. The combination of the germination rate and growth inhibition phenotype under salt stress was applied to determine the appropriate salt concentration.

### Determination of physiological parameters under salt stress during seed germination

Eleven sunflower seeds from each germplasm line were sown and treated with 300 mM NaCl (as treatment) and distilled water (as control), respectively. Four biological replicates were conducted for each treatment.

The number of germinated seeds was recorded every day for one week. On the seventh day after sowing, the root length (RL) and fresh weight (FW) of the seedlings were measured. The dry weight (DW) was determined after the seedlings were dried in a 150 °C-oven until they reached a constant weight.

To evaluate the salt tolerance of sunflower germplasms at the seed germination stage, germination rate (GR), germination index (GI), germination energy (GE), germination vigor index (GVI), and water content (WC) were calculated with the following formula, respectively:

$$\begin{array}{ccc}{\rm{GR}} & = & \frac{{G}_{7}}{N}\times 100 \% ;\\ {\rm{GI}} & = & \sum \frac{{G}_{t}}{T};\\ {\rm{GE}} & = & \frac{{G}_{1}}{N}\times 100 \% ;\\ {\rm{GVI}} & = & \sum \frac{{G}_{t}}{T}\times {\rm{AFW}};\\ {\rm{WC}} & = & \frac{FW-DW}{FW}\times 100 \% .\end{array}$$

In each of these formula, *T* is the number of days after sowing, *G*_*t*_ is the total number of germinated seeds on the *T*^th^ day, *G*_1_ and *G*_7_ are the total numbers of seed germinated on the 1^st^ and 7^th^ days after sowing, *N* is the total number of seeds, and AFW is the average FW of seedlings.

To describe the differences in salt tolerance among sunflower germplasms, the salt tolerance index (STI) of each physiological parameter was also measured.

$$ST{I}_{i}=\frac{{V}_{in}}{{V}_{ic}}$$

Here, STI_i_ is the STI of trait *i*, *V*_*i*n_ and *V*_*i*c_ represent the values of trait *i* in the salt-stressed treatment and control, respectively. Each trait of each germplasm has its own STI.

### Salt tolerance evaluation

The salt tolerance levels of sunflower germplasm lines were evaluated with the fuzzy comprehensive evaluation method using the membership function value (MFV)^21^. The salt tolerance MFV was calculated using the following equation:

$${X}_{i}=\frac{X-{X}_{min}}{{X}_{max}-{X}_{min}}\times 100 \% .$$

Here, *X*_*i*_ represents the membership function value of the *i* trait in a germplasm, *X* is the STI value of the *i* trait in the germplasm, *X*_max_ and *X*_min_ are the maximum and minimum values of the STI of the *i* trait observed in all germplasms, respectively. Therefore, each trait has its own MFV, ranging from 0 to 1.

According to a previously reported method^20^, the salt tolerance levels of sunflower germplasms were divided into five grades based on mean value ($\bar{X}$) and standard deviation (SD) of MFV: $(1){X}_{i}\ge \bar{X}+1.64SD$, highly salt-tolerant (HST); $(2)\bar{X}+1.64SD > {X}_{i}\ge \bar{X}+1SD$, salt-tolerant (ST); $(3)\bar{X}+1SD > {X}_{i}\ge \bar{X}-1SD$, moderately salt-tolerant (MST); $(4)\bar{X}-1SD > {X}_{i}\ge \bar{X}-1.64SD$, salt-sensitive (SS); $(5)\bar{X}-1.64SD > {X}_{i}$, highly-saltsensitive (HSS).

### Statistical analysis

SPSS 25 (IBM Corp., Armonk, NY, USA) was employed to perform multiple regression analysis on the mean MFV (the dependent *Y* variable) and STI value (the independent STI_i_ variable). The following mathematical evaluation model for salt tolerance was established: $$Y={\beta }_{GR}ST{I}_{GR}+{\beta }_{GI}ST{I}_{GI}+{\beta }_{GE}ST{I}_{GE}+{\beta }_{RL}ST{I}_{RL}+$$$${\beta }_{FW}ST{I}_{FW}+{\beta }_{MC}ST{I}_{MC}+\mu $$, where *Y* represents the mean MFV, β is a nonnormalized coefficient, and μ is a constant representing the random error term.

## Results

### Determination of salt stress concentration

**Figure 1:**
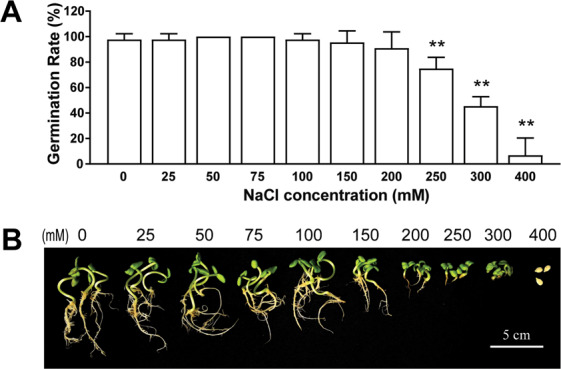
The optimum NaCl concentration for salt tolerance evaluation was determined according to the two indexes of germination rate and germination phenotype with the parental breeding line ZSADT. (**A**) Germination rate and (**B**) germination phenotype of ZSADT seeds treated with different NaCl concentrations for seven days. **Indicated significant difference (*p* < 0.01) between treatment and control.

To determine a suitable salt concentration for screening the salt tolerance of sunflower germplasms, ZSADT, a parental line, was used and the GR and germinated growth phenotype of ZSADT were recorded and photographed after 7 days of salt-stressed treatment (Fig. 1).

GR and seedling growth were significantly different among the different concentration treatments after 7 days (Fig. 1, *p* < 0.01). For low salinity treatments, up to 200 mM NaCl, the germination rate was not affected. NaCl concentrations above 200 mM significantly inhibited sunflower seed germination in a dose-dependent manner. At a NaCl concentration of 300 mM, more than half of seeds did not germinate, while the 400 mM NaCl treatment inhibited almost all seed germination, with only 6.8% final germinating (Fig. 1A). The phenotypic analysis under salt stress revealed that sunflower seedling growth and development after germination were more sensitive to salinity relative to seed germination. Although no significant growth inhibition were observed, when seedlings were exposed to NaCl concentrations up to 100 mM, 150 mM NaCl treatment significantly inhibited both leaf and root growth by day 7. Although the majority of seeds germinated when exposed to 200–300 mM NaCl, the growth of seedlings was almost completely inhibited (Fig. 1B). To ensure that biomass would permit measurements of indicators such as root length, fresh weight and water content as well, 300 mM NaCl was selected for evaluation of salt tolerance among sunflower germplasms at the germination stage.

### Effects of NaCl stress on plant traits of sunflower germplasm lines at the germination stage

Seeds from a total of 729 sunflower germplasm lines were harvested and made available. To ensure the accuracy of tests on salinity stress response, Pre-germination experiments were carried out to obtain the germplasm seeds with high vigor that the germination rate exceeded 99% for the screening of the salt tolerance, and finally, only 552 germplasm lines were candidates.

The STI value can be used to evaluate the effect of NaCl on the salt tolerance parameters of the sunflower germplasms. Larger STI values represent a smaller impact, while smaller values indicate greater impacts. The STI values under 300 mM NaCl for GR, GI, GE, RL, GVI, FW and WC of the 552 sunflower germplasm lines were shown in the supplementary material (Table S1). Under the 300 mM NaCl treatment, the GRs of four germplasm lines, 152021, 152094, 156084 and 156096, were significantly inhibited, but those of 10 other germplasms, including 151082, 152552, 152452 and 152400, were almost unaffected. Consistent with the GR results, the GI values of four germplasms, 152021, 152094, 156084 and 156096, were also significantly inhibited. GE was the most affected, with 328 germplasms having a GE of 0. The average STI value for GE among the 552 germplasms was only 0.062. The STI values of RL and GVI differed significantly among all germplasms. Because germination without cotyledon growth occurred, FW and WC of 27 germplasms couldn’t be measured. For 9 and 25 germplasms, FW and WC, respectively, were unaffected or slightly increased. Four germplasms, including 152021, did not germinate, and all parameters could not be measured, as they were apparently most affected by NaCl (Table S1).

### Salt tolerance evaluation

**Figure 2:**
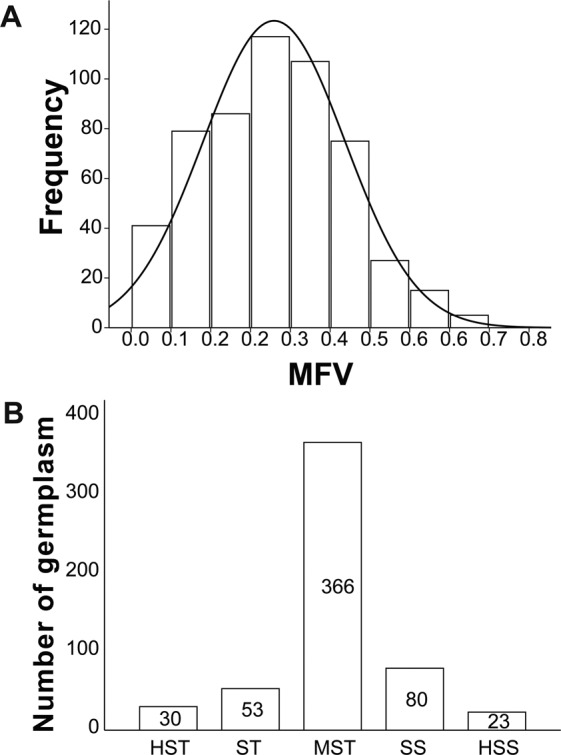
Classification of 552 sunflower germplasm lines based on mean membership function values (mean MFV). (**A**) Distribution of mean MFVs. (**B**) Classification of 552 sunflower germplasms according to salt tolerance based on mean MFVs. HST, highly salt-tolerant; ST, salt-tolerant; MST, moderately salt-tolerant; SS, salt-sensitive; HSS: highly salt-sensitive.

To comprehensively evaluate the salt tolerance of 552 germplasm lines, the MFV for each parameter of each germplasm and mean MFV were calculated (Table S2). The distribution of mean MFV is shown in Fig. 2A. The mean MFV ranged from 0.152 to 0.715 with an average of 0.287 ± 0.143. Germplasm line 505 had the highest mean MFV; four germplasm lines, 156084, 152021, 152094 and 156096, had the lowest mean MFV (0.000).

Based on the salinity response and salt tolerance, 552 tested sunflower germplasms were divided into five grades: (1) 30 germplasm lines were highly salt-tolerant (HST, ${X}_{i}\ge \bar{X}+1.64SD$, ${\rm{mean}}\,{\rm{MFV}}\,\ge \,0.5216$); (2) 53 were salt-tolerant (ST, $\bar{X}+1.64SD > {X}_{i}\ge \bar{X}+1SD$, 0.5216> mean MFV ≥0.4302); (3) 366 were moderately salt-tolerant (MST, $\bar{X}+1SD > {X}_{i}\ge \bar{X}-1SD$, $0.4302 > {\rm{mean}}\,{\rm{MFV}}\ge 0.1446$); (4) 80 were salt-sensitive (SS, $\bar{X}-1SD > {X}_{i}\ge \bar{X}-1.64SD$, $0.1446 > {\rm{mean}}\,{\rm{MFV}}\,\ge \,0.0532$); and (5) 23 were highly salt-sensitive (HSS, $\bar{X}-1.64SD\, > \,{X}_{i}$, 0.0532> mean MFV) (Fig. 2B, Table S2). The five most salt-tolerant and five most salt-sensitive germplasm lines were listed in Table 1. Based on this study, the majority of sunflower germplasm lines were moderately salt-tolerant; only a small proportion of germplasm lines were highly tolerant or highly sensitive to salinity.

**Table 1:** Five most salt-tolerant and five most salt-sensitive sunflower germplasm lines.

Germplasms	GR of MFV	GI of MFV	GE of MFV	RL of MFV	GVI of MFV	FW of MFV	WC of MFV	Mean MFV	Tolerance
152505	0.936	0.856	0.719	0.343	0.488	0.905	0.758	0.715	HST
156004	0.933	0.681	0.323	0.602	0.681	0.841	0.756	0.688	HST
156017	0.913	0.652	0.340	0.672	0.728	0.751	0.685	0.677	HST
151082	1.000	1.000	1.000	0.177	0.294	0.660	0.539	0.667	HST
151040	0.633	0.635	0.639	0.876	0.925	0.260	0.580	0.650	HST
152012	0.030	0.002	0.000	0.085	0.000	0.000	0.000	0.017	HSS
152021	0.000	0.000	0.000	0.000	0.000	0.000	0.000	0.000	HSS
152094	0.000	0.000	0.000	0.000	0.000	0.000	0.000	0.000	HSS
156084	0.000	0.000	0.000	0.000	0.000	0.000	0.000	0.000	HSS
156096	0.000	0.000	0.000	0.000	0.000	0.000	0.000	0.000	HSS

Germination rate (GR), germination index (GI), germination energy (GE), root length (RL), germination vigor index (GVI), fresh weight (FW) and water content (WC).

### Correlation analysis of parameter STI

The correlation coefficients between different parameters were analyzed (Table 2). Among all the parameters assessed, the correlation between GR and GI was the highest (*r* = 0.897), followed by FW and WC (*r* = 0.812). The correlation between RL and GE was the lowest (*r* = 0.192), followed by GE and WC (*r* = 0.214). The correlation between GE or RL and other parameters was also low.

**Table 2:** Correlation coefficients between mean membership function value (MFV) and parameter STI for 552 sunflower germplasms at the germination stage.

	STI_*GR*_	STI_*GI*_	STI_*GE*_	STI_*RL*_	STI_*GVI*_	STI_*FW*_	STI_*WC*_
STI_*GR*_	1	0.897	0.331	0.233	0.637	0.743	0.633
STI_*GI*_	0.897	1	0.641	0.268	0.739	0.64	0.552
STI_*GE*_	0.331	0.641	1	0.192	0.533	0.227	0.214
STI_*RL*_	0.233	0.268	0.192	1	0.73	0.371	0.316
STI_*GVI*_	0.637	0.739	0.533	0.73	1	0.584	0.497
STI_*FW*_	0.743	0.64	0.227	0.371	0.584	1	0.812
STI_*WC*_	0.633	0.552	0.214	0.316	0.497	0.812	1
Mean MFV	0.885	0.904	0.545	0.511	0.842	0.848	0.778

Salt tolerance index (STI) of germination rate (STI_*GR*_), germination index (STI_*GI*_), germination energy (STI_*GE*_), root length (STI_*RL*_), germination vitality index (STI_*GVI*_), fresh weight (STI_*FW*_) and water content (STI_*WC*_). These values are Pearson correlation coefficients (*r*).

**Figure 3:**
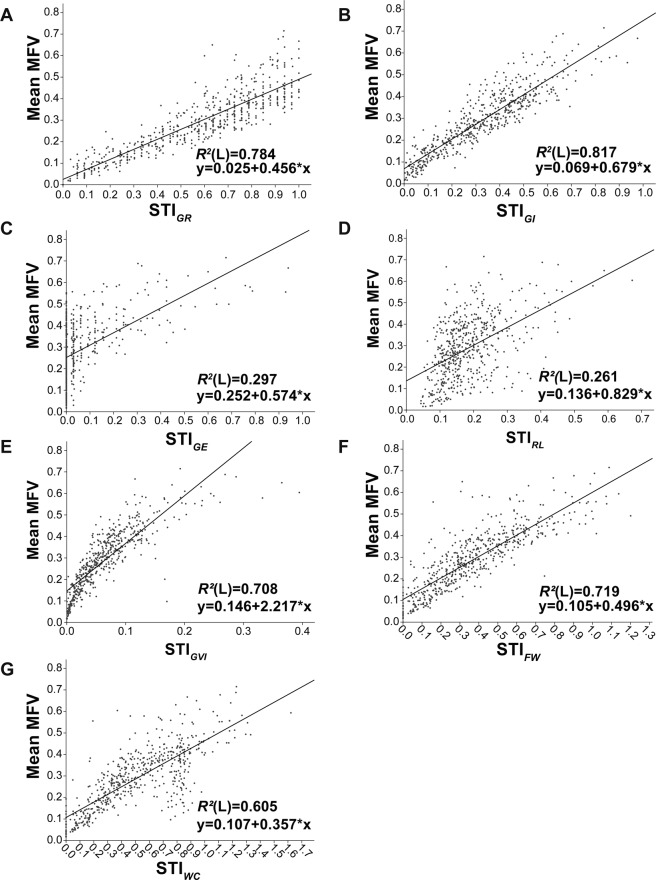
A linear correlation analysis between the STI values of each parameter and mean MFV. (**A**) Relationship between the STI of germination rate (STI_*GR*_) and mean MFV; (**B**) Relationship between the STI of germination index (STI_*GI*_) and mean MFV; (**C**) Relationship between the STI of the germination energy (STI_*GE*_) and mean MFV; (**D**) Relationship between STI of root length (STI_*RL*_) and mean MFV; (**E**) Relationship between STI of germination vitality index (STI_*GVI*_) and mean MFV; (**F**) Relationship between STI of fresh weight (STI_*FW*_) and mean MFV; (**G**) Relationship between the STI of water content (STI_*WC*_) and mean MFV; *R*^2^(L) is the coefficient of determination.

Mean MFV reflects the salt tolerance of germplasms. The larger the mean MFV of a germplasm is, the stronger its salt tolerance is. In this study, the STI of GR, GI, GE, RL, GVI, FW and WC together determined the mean MFV. Therefore, for each germplasm, the mean MFV value is determined by the STI of each parameter. To find the most reliable parameters reflecting salt tolerance, a linear model was fitted between each STI and mean MFV (Fig. 3). The coefficient of determination between the mean MFV and GI was the highest (*R*^2^ = 0.817), while those for GR, FW and GVI were slightly lower, (*R*^2^ = 0.784, *R*^2^ = 0.719 and *R*^2^ = 0.708, respectively. The coefficient of determination for RL was the lowest (*R*^2^ = 0.261). The normalized beta coefficients between mean MFV and GI as well as mean MFV and GVI were also higher. Overall, our results suggested that GI and GVI can be used as reliable traits to evaluate the salt tolerance of sunflower germplasm lines at the germination stage.

### Establishment of a model for evaluating salt tolerance in sunflower

Based on the mean MFV of 552 sunflower germplasm lines and STI values for seven parameters, a quantitative model for evaluating salt tolerance in sunflower was established; a linear equation was developed using multiple regression. As shown in Table 3, the unstandardized coefficients of the STI for GR, GI, GE, RL, GVI, FW and WC were 0.143, 0.146, 0.152, 0.213, 0.369, 0.119 and 0.088, respectively. The random error term was −3.33 × 10^−16^. Therefore, $Y=0.143\times {{\rm{STI}}}_{GR}+0.146\times {{\rm{STI}}}_{GI}+0.152\times {{\rm{STI}}}_{GE}+0.213\times {{\rm{STI}}}_{RL}+0.369\times {{\rm{STI}}}_{GVI}+0.119\times {{\rm{STI}}}_{FW}+0.088\times {{\rm{STI}}}_{WC}$

where *Y* represented the salt tolerance of sunflower germplasm lines. According to the classification criteria of this study, the salt tolerance of sunflower germplasms could be divided into five grades. When $Y\ge 0.5216$ indicated highly salt-tolerant (HST), $0.5216 > Y\ge 0.4302\,$indicated salt-tolerant (ST), $0.4302 > Y\ge 0.1446$ indicated moderately salt-tolerant (MST), $0.1446 > Y\ge 0.0532$ indicated salt-sensitive (SS), and *Y* < 0.0532 indicated highly salt-sensitive (HSS).

**Table 3:** Multiple linear regression analysis between mean MFV and salt tolerance index (STI) under 300 mM NaCl.

Model	Unstandardized coefficients		Standardized coefficients	*t*	Significance
	μ or β	SE	B		
Constant	−3.33 × 10^−16^	0.000		0.000	1.000
STI of GR	0.143	0.000	0.272	92395983.693	0.000
STI of GI	0.146	0.000	0.195	55437978.974	0.000
STI of GE	0.152	0.000	0.144	93652855.654	0.000
STI of RL	0.213	0.000	0.131	86814867.449	0.000
STI of GVI	0.362	0.000	0.137	63829921.170	0.000
STI of FW	0.119	0.000	0.203	121105524.259	0.000
STI of WC	0.088	0.000	0.192	136462357.506	0.000

Salt tolerance index (STI) of germination rate (STI of GR), germination index (STI of GI), germination energy (STI of GE), root length (STI of RL), germination vitality index (GVI), fresh weight (STI of FW) and water content (STI of WC). Mean MFV is the dependent variable, and β values are unstandardized coefficients. The constant µ represents the random error term.

To test whether the quantitative evaluation model was useful for predicting the salt tolerance of the sunflower germplasms, the *Y* values of the 552 germplasms were calculated (Table S3) and datawere used from three random germplasms of each of the five salt-tolerance grades, as a total of 15, listed in Table 4. The absolute value of the difference between *Y* and the mean MFV was also calculated (Table S3 and Table 4). The average difference between *Y* and mean MFV was only 0.000462, while the maximum and minimum were 0.002949 and 3.33 × 10^−16^, respectively. The values of mean MFV and *Y* were very close. Overall, our model was reliable and salt tolerance can be predicted by calculating the Y value of any sunflower germplasm using the STI values of growth parameters, such as GR, GI, GE, RL, GVI, FW and WC during the germination stage.

**Table 4:** Salt tolerance verification of multiple regression analysis with mean MFV.

Germplasm	STI_*GR*_	STI_*GI*_	STI_*GE*_	STI_*RL*_	STI_*GVI*_	STI_*FW*_	STI_*WC*_	Mean MFV	Y
152505	0.936	0.836	0.676	0.230	0.193	1.087	1.228	0.71509	0.71629
156004	0.933	0.665	0.303	0.404	0.269	1.010	1.225	0.68799	0.68987
156017	0.913	0.637	0.319	0.451	0.287	0.901	1.111	0.67721	0.67925
156008	0.741	0.479	0.154	0.424	0.203	0.602	0.763	0.5017	0.5032
152478	0.876	0.785	0.688	0.115	0.091	0.449	0.499	0.49922	0.49972
152533	0.758	0.686	0.636	0.175	0.120	0.517	0.588	0.49903	0.49977
152209	0.879	0.424	0.030	0.166	0.071	0.728	0.824	0.41223	0.41273
151156	0.970	0.607	0.061	0.101	0.061	0.535	0.771	0.41185	0.41222
152101	0.727	0.505	0.152	0.176	0.089	0.643	0.738	0.41182	0.4124
156023	0.289	0.143	0.024	0.152	0.022	0.105	0.124	0.12933	0.12953
151072	0.107	0.043	0.000	0.135	0.006	0.019	0.847	0.12925	0.12924
152044	0.364	0.182	0.030	0.108	0.020	0.060	0.071	0.12675	0.12693
152059	0.061	0.015	0.000	0.099	0.001	0.042	0.048	0.04172	0.04177
152069	0.091	0.022	0.000	0.113	0.003	0.000	0.000	0.04115	0.04122
152237	0.091	0.022	0.000	0.081	0.002	0.027	0.032	0.04016	0.04021

The number in the first column refered to the sunflower germplasm line, followed by the salt tolerance index (STI) values for germination rate (STI_*GR*_), germination index (STI_*GI*_), germination energy (STI_*GE*_), root length (STI_*RL*_) and germination vitality index (STI_*GVI*_), fresh weight (STI_*FW*_) and water content (STI_*WC*_). The regression formula was $Y=0.143\times {{\rm{STI}}}_{GR}+0.146\times {{\rm{STI}}}_{GI}+0.152\times {{\rm{STI}}}_{GE}+0.213\times {{\rm{STI}}}_{RL}+0.369\times {{\rm{STI}}}_{GVI}+0.119\times {{\rm{STI}}}_{FW}+0.088\times {{\rm{STI}}}_{WC}$, and mean MFV-*Y* is the difference between Mean MFV and Y.

## Discussion

Throughout their long-term evolution, plants have established complex mechanisms for responding to different environmental stresses, including salinity. However, different germplasms within the same species show different responses to the same stress owing to germplasms being grown under different environmental conditions and/or being bred for different specific purposes. The ability of a plant to resist salt stress varies widely among species and varieties^24^. Generally, sunflower has moderate salt tolerance, but little has been reported about its resistance to salt stress^25^. Specifically, different germplasms have shown significant differences in their growth parameters under NaCl stress. The different responses of germplasms or genotypes to salt stress have previously been reported in other plant species^26–28^. Seed germination is the first stage of plant growth, and crops are more susceptible to stress during the germination stage. Salt stress is one of the most serious stresses faced by crops, and salinity can cause significant decreases in seed germination rate. When plant is planted in saline-alkali soil, the germination of salt-sensitive germplasm is significantly inhibited, resulting in crop yield loss and even plant death^29,30^. One potential mechanism is high NaCl stress causing dysfunction in seed metabolism, further resulting in inhibition of seed germination^31^. Studies have shown that the size and shape of seeds^32^, KNO_3_ treatment^33^, antioxidant levels^34^ and gene expression^35^ can affect the salt tolerance of sunflower during germination. However, using a single feature to identify salt tolerance may be inadequate. The physiological and biochemical indexes related to salt tolerance can be used as criteria for the screening of salt tolerance. However, when more sunflower germplasms are used for screening, the process is cumbersome and time-consuming. In this study, a simple and efficient method of identifying salt tolerance through the effect of salt treatment on sunflower phenotypic characteristics was developed.

Salinity not only affects seed germination but also seedling growth and development. Thus, germination rate alone cannot accurately evaluate the salt tolerance of sunflower seed germination, other related traits should also be included involving in evaluating plant responses to salinity. In this study, we used multiple parameters to evaluate the salt tolerance of sunflower germplasms by mean MFV at the germination stage. Among the 552 sunflower germplasms, 83 showed salt tolerance, 366 showed moderate salt tolerance, and 103 showed salt sensitivity (Fig. 2, Table S2). The maximum mean MFV was 0.715, indicating that 300 mM NaCl has a greater impact on sunflower seed germination across germplasms. Compared with salt-tolerant germplasms, some germplasms showed lower mean MFV, indicating that these germplasms had lower salt tolerance at the germination stage. Salt stress causes osmotic damage and ion stress to plants, resulting in accumulation of reactive oxygen species^36^. The salt-tolerant germplasms screened in this study may have superior active oxygen scavenging capacity, synthesize more osmotic adjustment substances and/or have accumulated more inorganic ions to obtain higher salt tolerance^37,38^.

Salt stress damages plants, leading to a variety of physiological and biochemical changes. However, not all parameters are useful in salt tolerance screening. In hybrid breeding, the lack of an accurate and reliable salt tolerance evaluation parameter is one of the factors limiting the success rate of conventional breeding for salt tolerance^39^. To more efficiently determine the salt tolerance of sunflower germplasms during germination, it is necessary to identify some reliable traits as indicators of salt tolerance. In this study, GI and GVI are reliable traits for evaluating the salt tolerance of sunflower germplasms.

Moreover, when defining the salt tolerance of one or several sunflower germplasms, it is difficult to judge the salt tolerance of the germplasms without a large number of other germplasms for comparison. To evaluate the salt tolerance of sunflower germplasms easily and reliably, a mathematical formula was established by multiple regression analysis, and the salt tolerance of sunflower germplasms was evaluated by calculating the *Y* value. According to the calculated *Y* value, the salt tolerance of the germplasms can be divided into five grades. The larger the Y value is, the higher the salt tolerance is. This study is the first to establish a mathematical evaluation model for salt tolerance of sunflower germplasms at the seed germination stage, and this regression formula can be applied to screen the salt tolerance of other sunflower germplasm lines for mechanical exploration and breeding of salt tolerance of sunflower.

## Conclusion

GI and GVI are two reliable traits for evaluating the salt tolerance of sunflower germplasms under 300 mM NaCl treatment. A mathematical model was also developed to evaluate the salt tolerance at the germination stage. Based on the model, 552 sunflower germplasms were classified into five grades: 30 HST, 53 ST, 366 MST, 80 SS and 23 HSS. These results have important theoretical and practical values for the evaluation salt tolerance of sunflower germplasms and breeding new cultivars with high salt-tolerance as well as exploration of the mechanisms underlying salt tolerance.

## Supplementary information

Supplementary Table S1

Supplementary Table S2

Supplementary Table S3
